# Supplementary material for: Local chromosome context is a major determinant of crossover pathway biochemistry during budding yeast meiosis
Source: eLife. 2016 Nov 18;5:e19669. doi: 10.7554/eLife.19669 (PMC5222560; doi:10.7554/eLife.19669)
Supplement: Supplementary file 1. — DOI: http://dx.doi.org/10.7554/eLife.19669.017 [file elife-19669-supp1.pdf]

Supplementary File 1—Yeast strains

Strain	Genotype	Used in figures
MJL3617	*MATa ho::LYS2 lys2 leu2 nuc1Δ::LEU2 VMA1-103 trp1::hisG*<br>---- -------- ---- ---- ----------- -------- ----------<br>*MATα ho::LYS2 lys2 leu2 nuc1Δ::LEU2 VMA1-103 trp1::hisG*<br><br>*arg4Δ(eco47III-hpaI) ura3::Ty1 his4'-URA3-[arg4-VRS103]-'his4*<br>-------------------- --------- ------------------------------<br>*arg4Δ(eco47III-hpaI) ura3::Ty1 his4::natMX-[arg4-VRS]-KlTRP1*	S1, 2
MJL3627	*MATa ho::LYS2 lys2 leu2 nuc1Δ::LEU2 VMA1-103 trp1::hisG*<br>---- -------- ---- ---- ----------- -------- -----------<br>*MATα ho::LYS2 lys2 leu2 nuc1Δ::LEU2 VMA1-103 trp1::hisG*<br><br>*arg4Δ(eco47III-hpaI) ura3::Ty1-[arg4-VRS103]-URA3*<br>-------------------- -----------------------------<br>*arg4Δ(eco47III-hpaI) ura3::natMX-[arg4-VRS]-KlTRP1*	S1, 2
MJL3618	MJL3617 + *pCUP1-VDE-kanMX-pCUP1-CUP1*<br>--------------------------<br>*CUP1*	1, 2, S2, 3, S3-1, S3-2<br>4, S4-1, 5, S5-1
MJL3624	MJL3627 + *pCUP1-VDE-kanMX-pCUP1-CUP1*<br>--------------------------<br>*CUP1*	1, 2, S2, 3, S3-1, S3-2<br>4, S4-1, 5, S5-1
MJL3645	MJL3617 + *sae2Δ::hphMX*<br>------------<br>*sae2Δ::hphMX*	S1
MJL3643	MJL3627 + *sae2Δ::hphMX*<br>------------<br>*sae2Δ::hphMX*	S1

Strain	Genotype	Used in figures
MJL3670	MJL3617 + *pCUP1-VDE-hphMX-pCUP1-CUP1 mlh3Δ::kanMX*<br>-------------------------- ------------<br>*CUP1 mlh3Δ::kanMX*	3, S3-1, S3-2, 4, S4-1
MJL3669	MJL3627 + *pCUP1-VDE-hphMX-pCUP1-CUP1 mlh3Δ::kanMX*<br>-------------------------- -------------<br>*CUP1 mlh3Δ::kanMX*	3, S3-1, S3-2, 4, S4-1
MJL3729	MJL3617 + *pCUP1-VDE-hphMX-pCUP1-CUP1 yen1Δ::hphMX slx1Δ::natMX*<br>-------------------------- ------------ ------------<br>*CUP yen1Δ::hphMX slx1Δ::URA3*<br><br>*kanMX-pCLB2-3HA-MMS4*<br>--------------------<br>*kanMX-pCLB2-3HA-MMS4*	3, S3-1, S3-2, 4, S4-1
MJL3728	MJL3627 + *pCUP1-VDE-hphMX-pCUP1-CUP1 yen1Δ::hphMX slx1Δ::natMX*<br>-------------------------- ------------ ------------<br>*CUP yen1Δ::hphMX slx1Δ::URA3*<br><br>*kanMX-pCLB2-3HA-MMS4*<br>--------------------<br>*kanMX-pCLB2-3HA-MMS4*	3, S3-1, S3-2, 4, S4-1
MJL3726	MJL3617 + *pCUP1-VDE-hphMX-pCUP1-CUP1 mlh3Δ::kanMX*<br>-------------------------- ------------<br>*CUP1 mlh3Δ::kanMX*<br><br>*pCLB2-3HA-MMS4::kanMX slx1Δ::natMX yen1Δ::hphMX*<br>--------------------- ------------ ------------<br>*pCLB2-3HA-MMS4::kanMX slx1Δ::URA3 yen1Δ::hphMX*	3, S3-1, S3-2
MJL3725	MJL3627 + *pCUP1-VDE-hphMX-pCUP1-CUP1 mlh3Δ::kanMX*<br>-------------------------- ------------<br>*CUP1 mlh3Δ::kanMX*<br><br>*pCLB2-3HA-MMS4::kanMX slx1Δ::natMX yen1Δ::hphMX*<br>--------------------- ------------ ------------<br>*pCLB2-3HA-MMS4::kanMX slx1Δ::URA3 yen1Δ::hphMX*	3, S3-1, S3-2

Strain	Genotype	Used in figures
MJL3806	*pCUP1-VDE-kanMX-pCUP1-CUP1 pch2Δ::URA3*<br>MJL3617 + ------------------------- -----------<br>*pCUP1-CUP1 pch2Δ::URA3*	4, S4-1, S4-2
MJL3803	*pCUP1-VDE-kanMX-pCUP1-CUP1 pch2Δ::URA3*<br>MJL3627 + ------------------------- -----------<br>*pCUP1-CUP1 pch2Δ::URA3*	4, S4-1, S4-2
MJL3804	*pCUP1-VDE-kanMX-pCUP1-CUP1 pch2Δ::URA3 mlh3Δ::kanMX*<br>MJL3617 + ------------------------- ----------- ------------<br>*pCUP1-CUP1 pch2Δ::URA3 mlh3Δ::kanMX*	4, S4-1, S4-2
MJL3805	*pCUP1-VDE-kanMX-pCUP1-CUP1 pch2Δ::URA3 mlh3Δ::kanMX*<br>MJL3627 + ------------------------- ----------- ------------<br>*pCUP1-CUP1 pch2Δ::URA3 mlh3Δ::kanMX*	4, S4-1, S4-2
MJL3866	*pCUP1-VDE-kanMX-pCUP1-CUP1 pch2Δ::URA3 yen1Δ::hphMX*<br>MJL3617 + ------------------------- ----------- ------------<br>*pCUP1-CUP1 pch2Δ::URA3 yen1Δ::hphMX*<br><br>*slx1Δ::natMX pCLB2-3HA-MMS4::kanMX*<br>------------ --------------------<br>*slx1Δ::natMX pCLB2-3HA-MMS4::kanMX*	4, S4-1, S4-2
MJL3858	*pCUP1-VDE-kanMX-pCUP1-CUP1 pch2Δ::URA3 yen1Δ::hphMX*<br>MJL3627 + ------------------------- ----------- ------------<br>*pCUP1-CUP1 pch2Δ::URA3 yen1Δ::hphMX*<br><br>*slx1Δ::natMX pCLB2-3HA-MMS4::kanMX*<br>------------ --------------------<br>*slx1Δ::natMX pCLB2-3HA-MMS4::kanMX*	4, S4-1, S4-2
MJL3606	*pCUP1-VDE-kanMX-pCUP1-CUP1 spo11-Y135F-HA3-his6::kanMX*<br>MJL3617 + ------------------------- ---------------------------<br>*pCUP1-CUP1 spo11-Y135F-HA3-his6::kanMX*	5, S5-1, S5-2

Strain	Genotype	Used in figures
MJL3605	MJL3627 + *pCUP1-VDE-kanMX-pCUP1-CUP1* / *pCUP1-CUP1*; *spo11-Y135F-HA3-his6::kanMX* / *spo11-Y135F-HA3-his6::kanMX*<br>*pCUP1-VDE-kanMX-pCUP1-CUP1 spo11-Y135F-HA3-his6::kanMX*<br>-------------------------- ---------------------------<br>*pCUP1-CUP1 spo11-Y135F-HA3-his6::kanMX*	5, S5-1, S5-2
MJL3697	MJL3617 +<br>*pCUP1-VDE-hphMX-pCUP1-CUP1 mlh3Δ::kanMX*<br>-------------------------- -------------<br>*CUP1 mlh3Δ::kanMX*<br><br>*spo11-Y135F-HA3-his6::kanMX*<br>---------------------------<br>*spo11-Y135F-HA3-his6::kanMX*	5B, S5-1, S5-2
MJL3696	MJL3627 +<br>*pCUP1-VDE-hphMX-pCUP1-CUP1 mlh3Δ::kanMX*<br>-------------------------- -------------<br>*CUP1 mlh3Δ::kanMX*<br><br>*spo11-Y135F-HA3-his6::kanMX*<br>---------------------------<br>*spo11-Y135F-HA3-his6::kanMX*	5C, S5-1, S5-2
MJL3731	MJL3617 +<br>*pCUP1-VDE-hphMX-pCUP1-CUP1 yen1Δ::hphMX slx1Δ::natMX*<br>--------------------------- ------------ -------------<br>*CUP1 yen1Δ::hphMX slx1Δ::URA3*<br><br>*pCLB2-3HA-MMS4::kanMX spo11-Y135F-HA3-his6::kanMX*<br>--------------------- ---------------------------<br>*pCLB2-3HA-MMS4::kanMX spo11-Y135F-HA3-his6::kanMX*	5B, S5-1, S5-2
MJL3730	MJL3627 +<br>*pCUP1-VDE-hphMX-pCUP1-CUP1 yen1Δ::hphMX slx1Δ::natMX*<br>-------------------------- ------------ ------------<br>*CUP1 yen1Δ::hphMX slx1Δ::URA3*<br><br>*pCLB2-3HA-MMS4::kanMX spo11-Y135F-HA3-his6::kanMX*<br>--------------------- ---------------------------<br>*pCLB2-3HA-MMS4::kanMX spo11-Y135F-HA3-his6::kanMX*	5C, S5-1, S5-2

Supplementary File 1—Yeast Strains. The *ura3::Ty* allele contains a Ty element near a *Rsa*I site in *URA3* coding sequences (T.-C. Wu and M. Lichten, unpublished observations). *his4'-URA3-[arg4-VRS103]-'his4* and *ura3::Ty-[arg4-VRS103]-URA3* inserts are identical to inserts in MJL1077 and MJL1094 (Wu & Lichten 1995) except that they contain the *arg4-VRS103* allele (see Materials and Methods). In *his4::natMX-[arg4-VRS]-KlTRP1*, *natMX* sequences (Goldstein & McCusker 1999) replace sequences from chromosome V coordinate 66033 to +483bp downstream of *URA3* and *K. lactis TRP1* sequences (Stark & Milner 1989) replace sequences from +350bp downstream of *ARG4* to chromosome V coordinate 67716; in *ura3::natMX-[arg4-VRS]-KlTRP1*, *natMX* sequences (Goldstein & McCusker 1999) replace sequences from chromosome III coordinate 115943 to +483bp downstream of *URA3* and *K. lactis TRP1* sequences (Stark & Milner 1989) replace sequences from +356bp downstream of *ARG4* to chromosome III coordinate 116919 (all yeast genome coordinates from Saccharomyces Genome Database build #R64-2-1). *pCUP1-VDE-kanMX-pCUP1-CUP1* contains plasmid pMJ920 (see Materials and Methods) integrated at the *CUP1* locus; in *pCUP1-VDE-hphMX-pCUP1-CUP1*, the G418-resistance module is replaced by a hygromycin-resistance module (Goldstein & McCusker 1999). The following alleles have been previously described: *kanMX-pCLB2-3HA-MMS4* (Jessop et al. 2006); *spo11-Y135F-HA3-his6::kanMX* (Kee & Keeney 2002). *mlh3Δ::kanMX*, *slx1Δ::natMX* and *slx1Δ::URA3* and *yen1Δ::hphMX* were constructed by an exact replacement of the relevant coding sequences by the indicated drug-resistance module (Goldstein & McCusker 1999) or by *URA3* coding sequences. *pch2* mutant strain was constructed by replacing the wild type *PCH2* locus with the *pch2Δ::URA3* allele from pSS52 (San-Segundo & Roeder 1999) where the +266 to +1741 coding region of *PCH2* (*Acc*I-*Bam*HI fragment) has been replaced with a *URA3* fragment (*Cla*I-*Bam*HI).

References

Goldstein, A.L., McCusker, J.H., 1999. Three new dominant drug resistance cassettes for gene disruption in *Saccharomyces cerevisiae*. *Yeast*, 15, 1541–1553.

Jessop, L., Rockmill, B., Roeder, G.S., Lichten, M., 2006. Meiotic chromosome synapsis-promoting proteins antagonize the anti-crossover activity of Sgs1. *PLoS Genetics*, 2, e155.

Kee, K., Keeney, S., 2002. Functional interactions between *SPO11* and *REC102* during initiation of meiotic recombination in *Saccharomyces cerevisiae*. *Genetics*, 160, 111–122.

Saccharomyces Genome Database [WWW Document], URL http://www.yeastgenome.org.

San-Segundo, P.A., Roeder, G.S., 1999. Pch2 links chromatin silencing to meiotic checkpoint control. *Cell*, 97, 313–324.

Stark, M.J., Milner, J.S., 1989. Cloning and analysis of the *Kluyveromyces lactis TRP1* gene: a chromosomal locus flanked by genes encoding inorganic pyrophosphatase and histone H3. *Yeast*, 5, 35–50.

Wu, T.C., Lichten, M., 1995. Factors that affect the location and frequency of meiosis-induced double-strand breaks in *Saccharomyces cerevisiae*. *Genetics*, 140, 55–66.
